# High-Risk Opioid Prescribing and Nurse Practitioner Independence

**DOI:** 10.1001/jamahealthforum.2024.4544

**Published:** 2024-12-20

**Authors:** Lucas D. Cusimano, Nicole Maestas

**Affiliations:** 1 Geisel School of Medicine at Dartmouth, Hanover, New Hampshire; 2 Department of Health Care Policy, Harvard Medical School, Boston, Massachusetts

## Abstract

**Importance:**

Concerns around excessive opioid prescribing have been used to argue against the expansion of the scope of practice of nurse practitioners (NPs), but the association of NP practice independence with high-risk opioid prescribing is not well understood.

**Objective:**

To assess whether the rates of high-risk opioid prescribing changed in association with NP independence legislation.

**Design, Setting, and Participants:**

This difference-in-differences analysis compared rates of high-risk opioid prescribing in 6 states over 2 years following the adoption of NP independence compared with 10 neighboring nonadopting states from January 2012 to December 2021. Prescription insurance claims for 2 874 213 continuously enrolled individuals (members) aged 18 to 64 years from Blue Cross Blue Shield Axis were analyzed. Data analysis was carried out from 2021 to 2024.

**Exposure:**

Timing of the legislative effective date of NP independence in a state.

**Main Outcomes and Measures:**

The primary outcome was the rate of opioid prescriptions that overlapped with a prescription for a central nervous system (CNS) depressant. Secondary outcomes included the number of days of opioid-CNS depressant overlap, as well as the dosage and days supplied of opioids among all members and among opioid-naive members.

**Results:**

Six states that adopted NP independence legislation and 10 nonadopting neighboring states were similar in terms of demographic characteristics and had comparable pretrends in prescribing. The estimated change in the rate of opioid prescriptions that overlapped with a CNS depressant was −0.03 per 100 members per month (95% CI, −0.11 to 0.05). Changes in the number of days of opioid-CNS depressant overlap and in the dosage and days supplied of opioids among all members and among opioid-naive members were also small and statistically insignificant.

**Conclusions and Relevance:**

The results of this difference-in-differences analysis suggest that there was no relative increase in rates of high-risk opioid prescribing during the 2 years following the adoption of independence for NPs.

## Key Points

**Question:**

Were state policies that allowed nurse practitioners to work independently of physicians associated with rates of high-risk opioid prescribing?

**Findings:**

In this difference-in-differences analysis of opioid prescribing in 16 states, there was no change in the rates of high-risk opioid prescribing in the 6 states that adopted nurse practitioner independence compared with 10 nonadopting neighboring states during the 24 months following adoption.

**Meaning:**

The study found no association between legislation that granted independence to nurse practitioners and rates of risky opioid prescribing.

## Teaser

This difference-in-differences analysis examines whether the rates of high-risk opioid prescribing changed in association with nurse practitioner independence legislation.

## Introduction

In 2021, more than 1 in 5 opioid overdose deaths were attributed to prescription opioids in the US, and the rate of such deaths has increased 5-fold since 1999.^1^ To mitigate harms associated with opioid prescriptions, the US Centers for Disease Control and Prevention (CDC) published prescribing guidelines for opioids in 2016 and again in 2022.^2,3^ By 2014, 18 states and Washington DC had granted full practice independence for nurse practitioners (NPs) (that included prescribing of Schedule II narcotics), which removed all legally mandated physician supervision and collaboration requirements. Between 2014 and 2019, an additional 7 states granted this independence.^4^ Alongside these legislative changes, the share of all health care visits provided by NPs increased from 8.9% in 2013 to 17.3% in 2019.^5^

Proponents argued that practice independence for NPs would expand access to care and reduce costs, because NP services are typically billed at lower rates than physician services.^6^ However, there was concern by opponents that an expanded scope of NP practice might lead to more dangerous opioid prescribing, worsening the nationwide epidemic. In 2020, the American Medical Association opposed the proposed expansion of scope of practice for NPs in California, arguing that the legislation would not increase access to care or reduce costs and would lead to overprescribing, especially of opioids.^7^

Studies of practice independence legislation across states have found mixed associations with opioid prescribing. In a cross-sectional study comparing NPs in states with practice independence with those with more restrictive practice environments, NPs were more than 20 times more likely to exhibit risky opioid prescribing practices.^8^ Another cross-sectional examination of Medicare Part D claims found that NPs and physicians in states with a full NP scope of practice prescribed more oxycodone and hydrocodone compared with states with restricted NP practice rules.^9^ These cross-sectional studies did not analyze states before and after the legislation and thus could not account for higher base rates of opioid prescribing in some states. McMichael et al^10^ used a difference-in-differences approach and found that opioid prescribing by NPs and physicians to patients with commercial insurance declined after the adoption of NP scope of practice laws. Another study that used a difference-in-differences approach found that expanded scope of NP practice was associated with increased access to mental health services among the Medicaid population but did not change opioid prescribing in general.^11^

In addition, past research has used measures of opioid prescribing that could not distinguish between appropriate and inappropriate prescribing. Because opioids can serve legitimate medical purposes, our study focused on high-risk opioid prescribing. Previous studies typically did not account for substitution across clinician types, nor NPs billing under physician provider numbers, which is why we aggregated measures of prescribing. Additionally, previous studies used yearly data, which are coarse considering that legislation can go into effect throughout the year. We used monthly prescribing data, which allowed for more precise comparisons around the exact date that NPs gained independence. Lastly, our study compared states not only with themselves in the past, but with similar, neighboring states to eliminate state-specific factors and regional trends that were associated with opioid prescribing.

We investigated the associations of scope of practice legislation with opioid prescribing to commercially insured adults aged 18 to 64 years by all types of clinicians. We did not evaluate whether NPs prescribed opioids in ways that differ from physicians, but rather we measured aggregate changes following independence legislation.

## Methods

### Data

**Figure 1.**
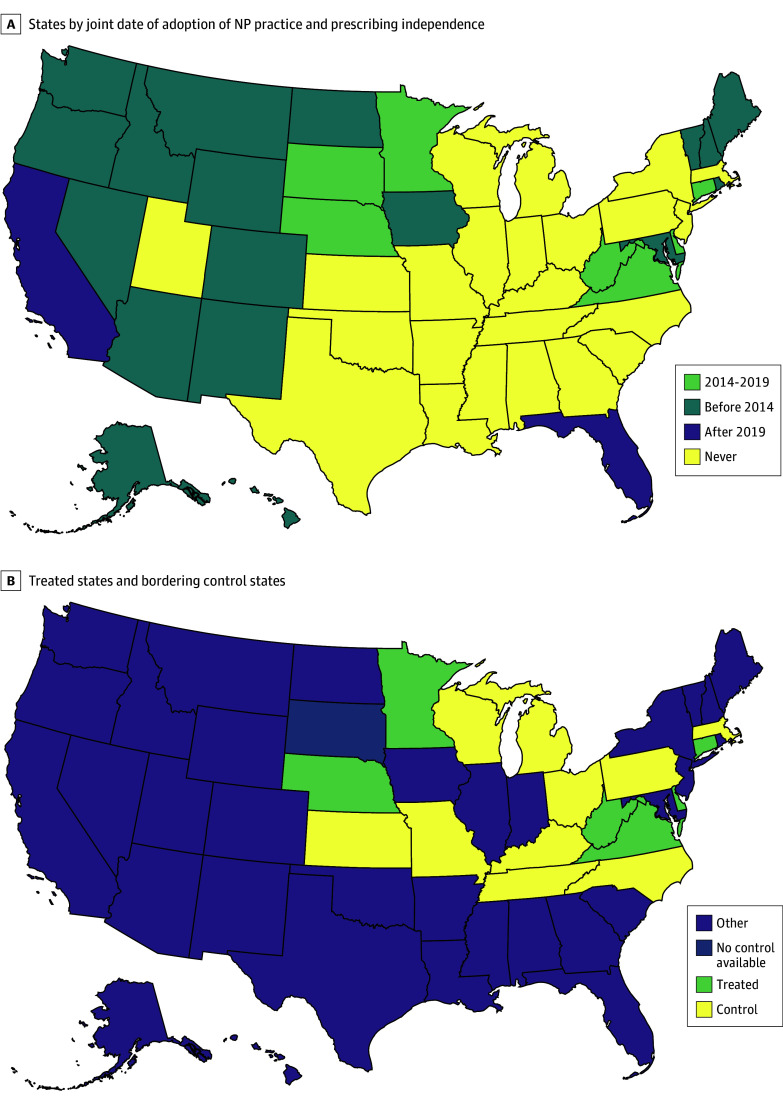
Classifications of States Into Treatment Periods and Bordering Controls A, States by joint date of adoption of nurse practitioner (NP) practice and prescribing independence. B, Treated states and bordering control states. The yellow states in panel B are a subset of the yellow states in panel A (ie, those which border a treated state [light green]). South Dakota in dark blue indicates that all bordering states had already changed their legislation and were not available as controls.

We used prescription claims from Blue Cross Blue Shield (BCBS) Axis, one of the largest datasets of commercial claims in the US for January 2012 through December 2021.^12,13,14^ Our analysis sample (described later) captured 2.9% of the civilian population aged 18 to 64 years in 2012, with variation across states (eFigure 1 in Supplement 1). We used these prescription claims to generate measures of opioid prescribing. To identify prescriptions for opioids, benzodiazepines, and other central nervous system (CNS) depressants, we obtained the relevant national drug codes from the CDC’s Opioid National Drug Code and Oral MME Conversion File and the US Food and Drug Administration’s National Drug Code Directory.^15,16^ We excluded opioids for opioid use disorder, gastrointestinal disease, or cough indications, as detailed in the data analysis appendices. The data for opioid prescribing across states were comparable with statistics published by the CDC (eFigure 2 in Supplement 1). Data on state geography and population were from the US Census Bureau,^17^ and relevant bordering states are shown in Figure 1B. The Institutional Review Board at Harvard University approved this study and waived the requirement for consent. This study adhered to the Strengthening the Reporting of Observational Studies in Epidemiology (STROBE) reporting guidelines.

### Exposure

To identify which states adopted NP practice and prescribing independence and when the legislation went into effect, we followed McMichael and Markowitz^10^ and present these classifications in Figure 1A. States in yellow never adopted such legislation, states in light green adopted legislation between 2014 and 2019, states in dark green adopted legislation before 2014, and states in purple adopted legislation after 2019. We focused our analysis on states that adopted independence legislation between 2014 and 2019 (Connecticut, Delaware, Minnesota, Nebraska, Virginia, and West Virginia) because this allowed for a 24-month preperiod during which to rule out the presence of differential pretrends that might bias the difference-in-differences estimates. Yellow states were control states and light green states were treated, while purple states were not included in the main analysis. South Dakota was indicated in dark blue and had no bordering control states, and so it was also not included in the main analysis. Data on county rurality and county adjacency were obtained from the US Census Bureau for 2010.^18^ Economic and employment data were obtained from the Bureau of Labor and Statistics.^19^

### Outcomes

We defined an opioid prescription as high risk if it overlapped with a benzodiazepine or other CNS depressant prescription (ie, CNS depressants) or if it exceeded 7 days’ supply or 50 morphine milligram equivalents (MME) per day.^3,20,21,22,23,24^ Opioid prescriptions that exceed 7 days’ supply or 50 MME per day have been identified as being associated with an increased risk of overdose^25^ and overdose death.^26^

To determine overlapping prescriptions, we estimated a prescription’s end date by adding the days supplied to the fill date. We then designated an opioid prescription as overlapping with a CNS depressant if the opioid prescription ended after the CNS depressant prescription began and the CNS depressant prescription ended after the opioid prescription began. This method followed previous literature.^21,27,28^

A state’s opioid prescribing rate was then calculated as the number of prescriptions of a given type during a given month divided by the number of members in the state-month multiplied by 100. Numerators were calculated separately for all members and opioid-naive members. Opioid-naive members were those who did not have a claim for an opioid prescription during the prior 6 months. We separately analyzed prescriptions for opioid-naive patients due to their elevated risks of becoming long-term opioid users,^29^ receiving high-risk prescriptions into the future,^30^ and misusing opioids.^31^

**Figure 2.**
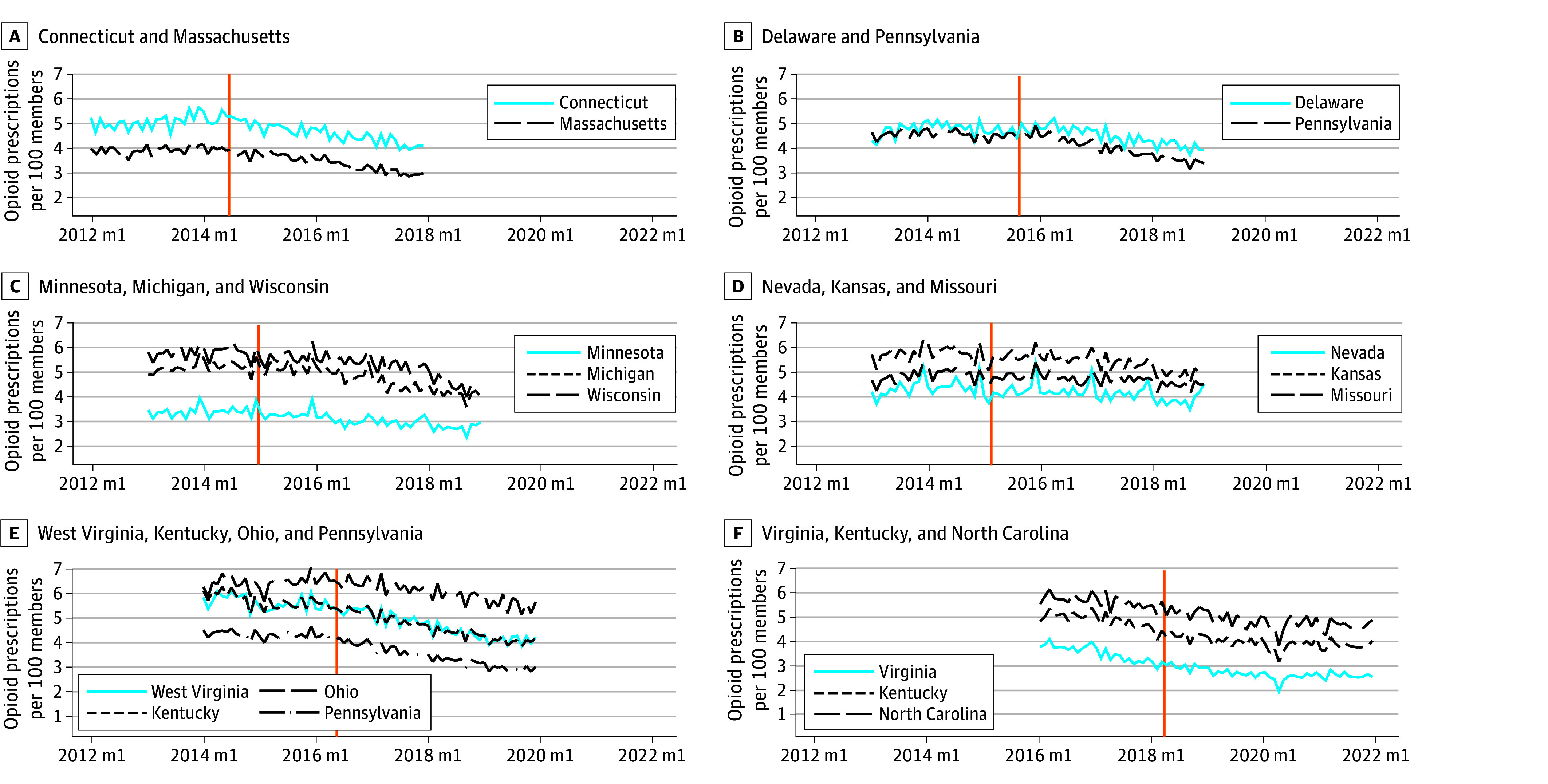
Rates of Opioid Prescribing in Treated and Control States in 6-Year Windows Around Nurse Practitioner (NP) Legislation Effective Date by Policy Cohort The solid blue line represents the treated state. The solid orange line represents the month of the legislation going into effect. The dotted and dashed lines are bordering control states. Tennessee was removed as a control group for Virginia due to a large unexplained decline in opioid prescribing. This drop could be explained by many factors, including changes in data reporting by Blue Cross Blue Shield plans.

The outcome set also included a state’s average days’ supply, average dosage strength (in MMEs per day), and the number of days of overlap per 100 members during each month. All prescriptions with more than 1000 units dispensed or with days supplied exceeding 365 days were dropped. Prescriptions with greater than 480 MME were top coded at 480 MME. These values, 1000 units dispensed and 480 MME, exceeded the 99.99th percentile of all opioid prescriptions. Aggregated BCBS Axis prescribing data are plotted by policy cohort in Figure 2.

### Study Design

We used a difference-in-differences method to compare the change in prescribing in states before and after the month that NP independence went into effect vs the change in states that had no such legislation during the same period. States with such legislation were considered treated, and those without such legislation were considered untreated and used as controls. Specifically, the primary criterion for a control state was that it did not adopt any practice independence or prescription independence for Schedule II drugs for NPs before 2020. Within this select group of states, we chose a state as a control if it bordered a treated state.

Neighboring states were chosen as controls because, empirically, they were similar to adopting states in terms of trends in prescribing before adopting NP legislation. In addition, we preferred control states to be ones that had a high chance of adopting NP legislation but ultimately did not. We observed that states tended to adopt NP legislation in geographic clusters, and so nonadopting neighboring states were more likely to be subject to similar legislative forces as those in adopting states.

The standard 2-way fixed-effects estimator is problematic when there are multiple periods and heterogenous treatment effects (eAppendix in Supplement 1).^32,33,34,35,36^ Instead, we implemented a stacked difference-in-differences design in which each treated geographic region, along with its assigned control regions, were considered a separate policy cohort.^37,38^ The primary regression analyses included state-by-cohort and month-by-cohort fixed effects and no additional covariates.

### Sample Construction

To create each policy cohort, we defined a 6-year sample window around the year that new NP legislation went into effect in the treated state. We then identified continuously enrolled members between ages 18 and 64 years who had primary medical and prescription drug coverage in every month during the sample window. This ensured that we could observe all claims for a balanced panel of members during the relevant period. We then identified opioid claims in the BCBS Axis pharmacy prescription table for these members. The eAppendix and eTable 1 in Supplement 1 provide additional details for each of the 6 cohorts.

Finally, because the policy variation occurred at the level of state and month, we collapsed the data to the state-month level for estimation. For each month and state, we summed prescription counts by prescription types, we summed days of overlap for risky prescription combinations and calculated the prescription-weighted average MME and days supplied. The state-month data for the policy cohorts were stacked in event time (ie, compared with the treatment month for the cohort) and analyzed using Stata/SE software, releases 16 and 18 (StataCorp).

### Robustness

To assess the robustness of our findings, we tested alternative specifications by varying our choices of control variables, patient samples, control states, and geographic levels of analysis. We tested a specification that controlled for state-level economic conditions, the number of physicians per population, and the number of NPs per population, as well as versions without certain sample restrictions. We separately analyzed prescribing rates for patients who, during the 2 years before the legislation change, either had an office visit with an NP or only saw physicians. Instead of using neighboring states as controls, we also used distance cutoffs based on centers of population and geography to include or exclude control states, as well as using all available control groups. We tested a version on the county level, rather than state level, for which we restricted to counties on the borders of states. In this case, aggregation was conducted at the county-month rather than state-month level. Lastly, we included controls for the presence of Prescription Drug Monitoring Program legislation.

## Results

### Balance on Characteristics

**Figure 3.**
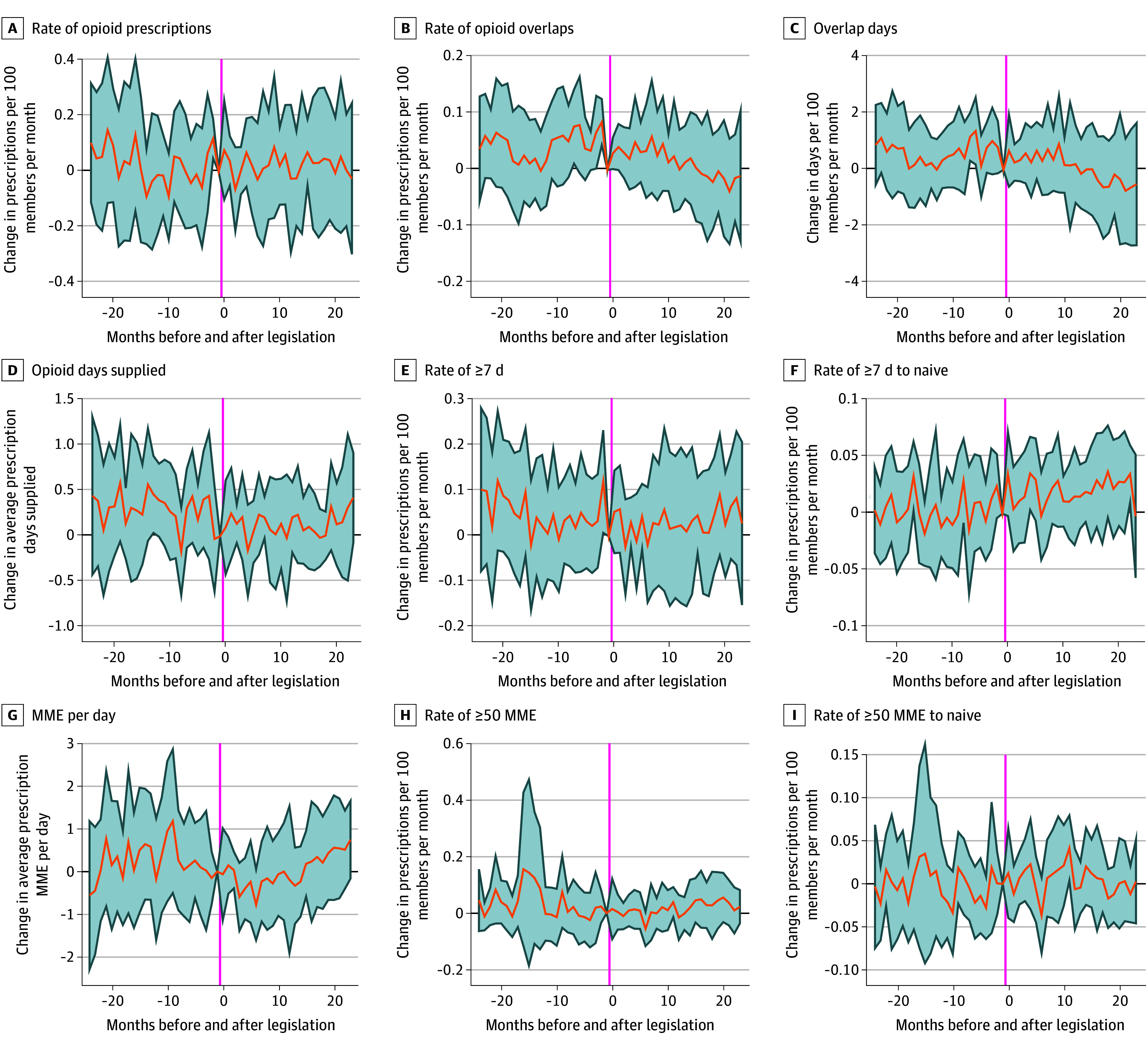
Relative Opioid Prescription Rates for Treated vs Control States In each panel, the y-axis measures the difference in the indicated opioid prescribing outcome in treatment vs control states, as estimated using equation 2. The x-axis measures event time, in which 0 is the legislation enactment month. The orange line plots the δj coefficients from equation 2 of the eAppendix in Supplement 1, and the gray shading is 95% CIs. Rates are prescriptions per 100 members per month. Overlap refers to an opioid prescription that overlapped with a central nervous system depressant prescription. MME indicates morphine milligram equivalents.

States that granted NPs practice and prescribing independence between 2014 and 2019 had similar characteristics 2 years before the legislation was passed as states that acted as controls, as shown in Table 1. Trends in outcomes were also similar before the legislation changes, as evidenced by Figure 3.

**Table 1.** Summary Statistics of States by NP Legislation Status

Variable	24 to 13 mo Before legislation^a^				13 to 24 mo After legislation^a^			
	State average		Difference	*P* value	State average		Difference	*P* value
	Treated	Control			Treated	Control		
Rural population, % (2010 US Census)	29	27	2	.73	Unchanged	NA	NA	NA
Unemployment, %	5.3	6.1	−0.7	.24	4.3	4.6	−0.3	.26
Mean annual pay, 2017 $	51 265	49 611	1654	.47	53 184	51 527	1657	.49
GP/family physicians per 100 000 population	43	44	0	.99	42	38	4	.69
NPs per 100 000 population	52	48	5	.49	63	65	−2	.80
Female individuals in the sample, %	51.8	51.2	0.5	.27	Unchanged	NA	NA	NA
Sample as % of civilian population aged 18-64 y (2010 US Census)	3.2	3.3	−0.1	.90	Unchanged	NA	NA	NA
Mean age at start of window, y	43.8	43.5	0.3	.25	Unchanged	NA	NA	NA
Opioid prescriptions per 100 members	4.57	5.24	−0.67	.18	3.9	4.4	−0.51	.31

Abbreviations: GP, general practitioner; NA, not applicable; NP, nurse practitioner.

^a^ Table reports coefficients when regressing variables on a binary ever-treated state variable with month-by-cohort fixed effects and clustering at the state level. Data are restricted to between 24 and 13 months before the treatment month of the relevant cohort. Unchanged indicates that, by definition, the data are unchanged from one period to the next.

### Prescribing Outcomes

We found that there was no change in the aggregate levels of any measure of opioid prescribing. As seen in Table 2, point estimates for changes in opioid prescribing were precisely estimated, small, and statistically insignificant.

**Table 2.** Difference-in-Differences Estimates of Opioid Prescription Outcomes Around Nurse Practitioner Independence

Characteristic	Opioid prescriptions					More than 7-d supply		More than 50 MME per d	
	All	Overlap	Overlap days	Days’ supply	MMEs	All	Naive	All	Naive
Legislation (SE) [95% CI]^a^	0.00 (0.06) [−0.12 to 0.12]	−0.03 (0.04) [−0.11 to 0.05]	−0.52 (0.72) [−2.07 to 1.04]	−0.16 (0.10) [−0.37 to 0.06]	−0.22 (0.29) [−0.83 to 0.39]	−0.02 (0.05) [−0.12 to 0.09]	0.02 (0.01) [−0.01 to 0.04]	−0.02 (0.03) [−0.09 to 0.05]	0.01 (0.01) [−0.02 to 0.03]
Outcome average	4.9	0.7	10.9	16.0	43.7	2.8	0.3	1.1	0.3
Change from average, %	0	−4	−5	−1	−1	−1	6	−2	2
Observations, No.	816	816	816	816	816	816	816	816	816
Adjusted *R*^2^	1.0	1.0	1.0	1.0	1.0	1.0	0.9	0.9	0.8
*F*	0.0	0.7	0.5	2.4	0.6	0.1	2.3	0.3	0.1

Abbreviation: MME, morphine milligram equivalents.

^a^ Standard errors in parentheses, clustered at the state level; state-by-cohort and month-by-cohort fixed effects.

The estimated change in the primary outcome, the rate of opioid prescriptions overlapping with a CNS depressant, was −0.03 prescriptions per 100 members per month (95% CI, −0.11 to 0.05) relative to a mean of 0.7 prescriptions per 100 members per month. Similarly, the change in the number of days of overlap was −0.52 (95% CI, −2.07 to 1.04). The changes in mean days’ supply and MMEs of prescriptions given were −0.16 (95% CI, −0.37 to 0.06) and −0.22 (95% CI, −0.83 to 0.39), respectively. Overall, the change in the rate of opioid prescribing was 0.00 per 100 members per month (95% CI, −0.12 to 0.12) relative to a mean of 4.9 prescriptions per 100 members per month.

The rate of prescriptions exceeding 7 days’ supply or 50 MME per day did not significantly change for either all members or opioid-naive members. The estimated change in the rate of prescriptions exceeding 7 days’ supply was −0.02 (95% CI, −0.12 to 0.09) for all members relative to a mean of 2.8 prescriptions per 100 members per month. The change in the rate of prescriptions exceeding 50 MMEs per day was also statistically insignificant at −0.02 (95% CI, −0.09 to 0.05) prescriptions per 100 members per month.

### Robustness

Specifications for robustness did not meaningfully change the results. Results for specifications with covariates are in eTable 2 in Supplement 1; results for the specification separated by patients who were exposed to NPs are in eTable 3 in Supplement 1; specifications for alternative control groups are in eTable 4 in Supplement 1; county-level analyses are in eFigure 3 and eTable 5 in Supplement 1; and results that controlled for the presence of Prescription Drug Monitoring Program legislation are in eTable 6 in Supplement 1.

## Discussion

In this difference-in-difference analysis, we examined changes in aggregate opioid prescribing around legislation allowing NPs to practice and prescribe independently of physicians. We used one of the largest available datasets of commercial insurance claims to analyze associations across the US over a 10-year period. Unlike previous studies of Medicare Part D claims, our analysis included middle-aged individuals, an important group who have been especially affected by the opioid epidemic. One advantage of our methods was that we created measures of opioid prescribing by aggregating claims data from a fixed, continuously enrolled sample of individuals. Accordingly, all comparisons were made between the same individuals living in the same location across years, ensuring our results were not driven by entry into or exit from the sample, or movement of patients across states. Moreover, we observed individuals even if they were not prescribed any opioids, and we determined if individuals were opioid naive (ie, they had not received an opioid during the previous 6 months).

We found that state legislation granting independence to NPs was not associated with changes in rates of high-risk opioid prescribing in the state. This finding was consistent across several measures, including concurrent opioid and CNS depressant prescribing and high-risk prescribing to opioid-naive patients.

One potential explanation for our precisely estimated, null findings is that NP independence may have reduced the administrative burden of complying with supervision and collaboration requirements but otherwise did not meaningfully change the day-to-day activities of NPs. NPs may have continued to consult with or refer patients to physicians to ensure appropriate opioid prescribing. If this was the case, reduced administrative burdens may have enhanced efficiency for clinicians and reduced health care costs. However, the study results imply that oversight requirements were not associated with reductions in risky opioid prescribing. When requirements were removed, there was no change in risky prescribing. Physicians themselves may not have been careful in their oversight duties, or they may have reduced their own risky prescribing to offset increases in risky prescribing by NPs following the legislation change.

Favorable financial reimbursement for NPs when billing under physician codes may have also encouraged continued collaboration even when not required by law. Although some recent evidence suggests that, cross-sectionally, NPs prescribed high-risk opioids at higher rates than physicians, we showed that relaxing physician supervision requirements on NPs was not associated with changes in high-risk opioid prescribing on net.

### Limitations

This study had limitations. First, legislation and practice patterns around opioid prescribing were changing throughout the 2010s, and so it is difficult to ascribe changes in rates of prescribing, or lack thereof, to any one type of legislation. However, the fact that we observed parallel trends in neighboring states and used variation around the month rather than year of the legislation change potentially assuages some of these concerns. Second, while the 6-year continuous enrollment requirement allowed us to produce a balanced panel of commercially insured members who did not change in composition over time, it could have also introduced selection bias. Individuals adversely affected by changes in opioid prescribing practices may have been more likely to drop out of the sample. For instance, if an individual received high-risk opioids, developed a substance use disorder, and subsequently lost their employment and/or their employer-sponsored health insurance coverage, they would have been excluded from the sample. Third, there may have been legislative endogeneity, in which states that anticipate changes in prescribing adjust the legislative environment in response. Fourth, there may have been spillovers across states (eg, if prescribers move to regions based on legislation). That said, the restrictions on member location allowed us to rule out patients who moved across state lines. Fifth, in terms of external validity, the study samples were limited to members who had health insurance coverage through BCBS plans, but there may have been changes in prescribing among other groups that we did not observe. Lastly, we investigated changes in prescribing up to 2 years after a change in legislation. The existing pool of NPs would have had experience working collaboratively or under the supervision of a physician. Newer cohorts of NPs would not have had this experience but would make up a growing portion of the workforce. Any effects of the change in legislation might appear more than 24 months later.

## Conclusions

This difference-in-difference analysis found that, following states granting independence to nurse practitioners, increases in high-risk opioid prescribing were not observed. Opioid prescribing is likely not a key factor in the safety of this type of legislation, as previously implemented by states. Future research may examine nuance in state policies around NPs (eg, those that did not extend NP independence to opioid prescribing).

## Notes

Supplement 1. eAppendix. eFigure 1. Percent of Total State Population Covered by Final BCBS Axis Analysis Sample eFigure 2. Validation of BCBS Opioid Prescribing Data with CDC Opioid Prescribing Data eFigure 3. Classification of Counties into Bordering Controls eFigure 4. Examples of Opioid Prescribing Data for County-level Cohorts eFigure 5. Timeline of PDMP Must-Access Laws Relative to NP Legislation eTable 1. Construction of Cohorts with Member Counts and Observation Counts eTable 2. Difference-in-Differences Estimated using Alternative Model Specifications eTable 3. Difference-in-Differences Estimates of Opioid Prescription Outcomes around NP Independence Split by NP Exposure eTable 4. Difference-in-Differences Estimated using Alternative Control Groups eTable 5. Difference-in-Differences Estimates of Opioid Prescription Outcomes around NP Independence with County Experiments eTable 6. Difference-in-Differences Estimates of Opioid Prescription Outcomes around NP Independence Controlling for Must-Access PDMP Legislation eReferences.

Supplement 2. Data sharing statement
