# Supplementary material for: High-Risk Opioid Prescribing and Nurse Practitioner Independence
Source: JAMA Health Forum. 2024 Dec 20;5(12):e244544. doi: 10.1001/jamahealthforum.2024.4544 (PMC11662257; doi:10.1001/jamahealthforum.2024.4544)
Supplement: Supplement 1. — eAppendix. eFigure 1. Percent of Total State Population Covered by Final BCBS Axis Analysis Sample eFigure 2. Validation of BCBS Opioid Prescribing Data with CDC Opioid Prescribing Data eFigure 3. Classification of Counties into Bordering Controls eFigure 4. Examples of Opioid Prescribing Data for County-level Cohorts eFigure 5. Timeline of PDMP Must-Access Laws Relative to NP Legislation eTable 1. Construction of Cohorts with Member Counts and Observation Counts eTable 2. Difference-in-Differences Estimated using Alternative Model Specifications eTable 3. Difference-in-Differences Estimates of Opioid Prescription Outcomes around NP Independence Split by NP Exposure eTable 4. Difference-in-Differences Estimated using Alternative Control Groups eTable 5. Difference-in-Differences Estimates of Opioid Prescription Outcomes around NP Independence with County Experiments eTable 6. Difference-in-Differences Estimates of Opioid Prescription Outcomes around NP Independence Controlling for Must-Access PDMP Legislation eReferences. [file jamahealthforum-e244544-s001.pdf]

Supplemental Online Content

Cusimano LD, Maestas N. High-risk opioid prescribing and nurse practitioner independence. *JAMA Health Forum*. 2024;5(12):e244544. doi:10.1001/jamahealthforum.2024.4544

eAppendix.

eFigure 1. Percent of Total State Population Covered by Final BCBS Axis Analysis Sample

eFigure 2. Validation of BCBS Opioid Prescribing Data with CDC Opioid Prescribing Data

eFigure 3. Classification of Counties into Bordering Controls

eFigure 4. Examples of Opioid Prescribing Data for County-level Cohorts

eFigure 5. Timeline of PDMP Must-Access Laws Relative to NP Legislation

eTable 1. Construction of Cohorts with Member Counts and Observation Counts

eTable 2. Difference-in-Differences Estimated using Alternative Model Specifications

eTable 3. Difference-in-Differences Estimates of Opioid Prescription Outcomes around NP Independence Split by NP Exposure

eTable 4. Difference-in-Differences Estimated using Alternative Control Groups

eTable 5. Difference-in-Differences Estimates of Opioid Prescription Outcomes around NP Independence with County Cohorts

eTable 6. Difference-in-Differences Estimates of Opioid Prescription Outcomes around NP Independence Controlling for Must-Access PDMP Legislation

eReferences.

This supplemental material has been provided by the authors to give readers additional information about their work.

© 2024 Cusimano LD et al. *JAMA Health Forum.*

eAppendix.

Data Analysis Appendices

Opioid Identification Strategy

We identified opioid prescriptions in the commercial pharmacy claims data by creating a list of NDCs for opioid drugs and matching this list to claims. We created the list of NDCs by combining two sources: the FDA's NDC database files[1] and the CDC's NDC and Oral MME Conversion file.[2]

We downloaded the NDC database file on November 2, 2021 and extracted "package.txt" and "product.txt." We did the same for the excluded drugs file. We merged the excluded drug files together and kept all entries containing the following strings in the proprietary name field:

<u>Determine if drug acts on opioid receptors</u>

Contains any of	and	Does NOT Contain
HYDROCOD		APOMORPHINE
BUPRENORPHINE		
BUTORPHANOL		
CODEINE		
DIHYDROCODEINE		
FENTANYL		
HYDROMORPHONE		
LEVORPHANOL		
MEPERIDINE		
METHADONE		
MORPHINE		
NALOXONE		
OXYCODONE		
OXYMORPHONE		
PENTAZOCINE		
TAPENTADOL		
TRAMADOL		

Relevant columns in CDC and FDA datasets:

	CDC	FDA
Name	PRODNME<br>GENNME<br>Drug	proprietaryname<br>nonproprietaryname<br>substancename
Delivery	Master_Form	packagedescription<br>dosageformname<br>routename
Strength	LongShortActing<br>DEAClassCode<br>Strength_Per_Unit	active_numerator_strength<br>active_ingred_unit<br>deaschedule<br>UOM<br>MME_Conversion_Factor
Drug Class		pharm_classes

© 2024 Cusimano LD et al. *JAMA Health Forum.*

Then we merged the package and product files from the “all drugs” database, and searched for the following terms:

Contains any of:	in:
OPIOID	Drug Class
HYDROCODONE	Name
CODEINE	Name
TRAMADOL	Name
PENTAZOCINE	Name

Does NOT contain any of:	in:
ANTICHOL	Drug Class
ANTIDIAR	Drug Class
ANTIHISTAM	Drug Class
EMETIC	Drug Class
EXPECT	Drug Class
SYMPATHOMIMET	Drug Class
VASODILAT	Drug Class
MUSCLE RELAX	Drug Class
SMOOTH-RESPIR	Drug Class

Determine whether drug is used for in-patients or outpatients

Injectables:

Contains any of:	in:
ALFENTANIL	Name
SUFENTANIL	Name
DEZOCINE	Name
REMIFENTANIL	Name
APOMORPHINE	Name
HEXAFLUORENIUM	Name
ALPHARODINE	Name

Other Delivery Methods:

Contains any of:	in:
POWDER	Delivery
TINC	Delivery
INJ	Delivery
SOLUTION and BUPRENORPH(IE)NE	Delivery and Name
TINCTURE and OPIUM	Delivery and Name
TOPICAL and TRAMADOL	Delivery and Name

Opioid for cough

Contains any of:	in:
PHENYLEPHRINE	Name
PROMETHAZINE	Name
GUAIFENESIN	Name
PSEUDOPHEDRINE	Name
HOMATROPINE	Name
CHLORPH(EI)N(IE)RAMINE	Name
HISTAMINE	Drug Class

Opioid use disorder

Contains any of:	in:
METHADONE	Name
BUPRENORPHINE	Name

© 2024 Cusimano LD et al. *JAMA Health Forum.*

LEVOMETHADYL	Name
LEVACETYLMETHADOL	Name
NALTREXONE	Name
NALOXONE	Name

Miscellaneous and Non-pain Opioid Agonists/Antagonists

Contains any of:	in:
LOPERAMIDE	Name
IMODIUM	Name
ELUXADOLINE	Name
NALOXEGOL	Name
ALVIMOPAN	Name
NALDEMEDINE	Name
METHYLNALTREXONE	Name
OLANZAPINE	Name
SAMIDORPHAN L-MALATE	Name

Benzodiazepines and CNS Depressant Identification
Benzodiazepines and other central-nervous system depressants which are dangerous when combined with opioids are identified using drug names from FDA data.[3]

**Generic Name**	**Drug Class**
alprazolam	Benzodiazepine
chlordiazepoxide	Benzodiazepine
clobazam	Benzodiazepine
clonazepam	Benzodiazepine
clorazepate	Benzodiazepine
diazepam	Benzodiazepine
estazolam	Benzodiazepine
flurazepam	Benzodiazepine
lorazepam	Benzodiazepine
oxazepam	Benzodiazepine
quazepam	Benzodiazepine
temazepam	Benzodiazepine
triazolam	Benzodiazepine
butabarbital	Other Sleep Drug/Tranquilizer
eszopiclone	Other Sleep Drug/Tranquilizer
pentobarbital	Other Sleep Drug/Tranquilizer
ramelteon	Other Sleep Drug/Tranquilizer
secobarbital	Other Sleep Drug/Tranquilizer
suvorexant	Other Sleep Drug/Tranquilizer
zaleplon	Other Sleep Drug/Tranquilizer
zolpidem	Other Sleep Drug/Tranquilizer
baclofen	Muscle Relaxant
carisoprodol	Muscle Relaxant
chlorzoxazone	Muscle Relaxant
cyclobenzaprine	Muscle Relaxant
dantrolene	Muscle Relaxant
metaxalone	Muscle Relaxant
methocarbamol	Muscle Relaxant
orphenadrine	Muscle Relaxant

© 2024 Cusimano LD et al. *JAMA Health Forum.*

tizanidine	Muscle Relaxant
aripiprazole	Antipsychotics
asenapine	Antipsychotics
cariprazine	Antipsychotics
chlorpromazine	Antipsychotics
clozapine	Antipsychotics
fluphenazine	Antipsychotics
haloperidol	Antipsychotics
iloperidone	Antipsychotics
loxapine	Antipsychotics
lurasidone	Antipsychotics
molindone	Antipsychotics
olanzapine	Antipsychotics
paliperidone	Antipsychotics
perphenazine	Antipsychotics
pimavanserin	Antipsychotics
quetiapine	Antipsychotics
risperidone	Antipsychotics
thioridazine	Antipsychotics
thiothixene	Antipsychotics
trifluoperazine	Antipsychotics
ziprasidone	Antipsychotics

Statistical Methods

For our application, we created a new data set for every state with a legislation change. Within that data set, there were 6 years of monthly data for the treated state and the relevant control states around the month that the treated state's legislation went into effect. This process was completed for each treated state, and then the data sets were appended together (i.e., "stacked"). This methodology was used to avoid comparisons that include already-treated states in the control group, which biases the standard two-way fixed effects estimator.[4] In addition, with heterogenous treatment effects, the standard method inappropriately weights treatment effects from each cohort.[4] It can apply negative weights to certain cohorts and overweight cohorts which contained a treatment date toward the beginning and end of the sample.[4]

The following model was estimated on the stacked data at the state-month-cohort level, with 24 months of observations before and after the month of treatment for each state:

$$\mathrm{Y}_{sti} = \delta \times \mathbf{1}(t \geq T_i \cap s \in S_i) + e_i \times \alpha_s + e_i \times \gamma_t + \varepsilon_{sti} \qquad (1)$$

where Y_{sti} represents a prescribing outcome (defined above) for state *s* in month *t* in cohort *i*. T_i is the date when NP legislation takes effect in the treated state in cohort *i*, while S_i defines the set of treated states in cohort *i*. Equation (1) includes state-by-cohort fixed effects, $e_i \times \alpha_s$, in order to constrain comparisons of treated and control states to only those states assigned to the same cohort, and calendar month-by-cohort fixed effects, $e_i \times \gamma_t$, to control for monthly trends affecting all states in a given cohort. The coefficient of interest, δ, is the difference between treated and control states after the legislation less the difference between such states before the legislation. Equation (1) was estimated using ordinary least squares regression, and standard errors were clustered at the state level.

A key assumption to interpret δ as the causal effect of the legislation is that, in the absence of the legislation, the outcome variable in control states would have continued in a parallel trajectory with those states that did pass the legislation. We must also assume that legislation in treated states does not affect outcomes before it becomes effective and does not affect other states through spillovers. To check whether treated and control states had parallel trajectories prior to the legislation, we estimated the following "event-study" model using 24 months of observations prior to and 24 months of observations following the legislation change. We then tested whether the $\hat{\delta}_j$ coefficients were statistically different from zero:

© 2024 Cusimano LD et al. *JAMA Health Forum.*

$$Y_{sti} = \sum_{j=-24}^{23} \delta_j \times \mathbf{1}(t = j \cap s \in S_i) + e_i \times \alpha_s + e_i \times \gamma_t + \varepsilon_{sti} \qquad (2)$$

Sample Construction

To assign members to states, we started with locations reported on opioid prescriptions filled during the relevant sample window. If multiple states or counties were reported across prescriptions for a given member, we excluded the member from the sample.[i] For members with no prescriptions during the window, we required that they received a professional service and used the location reported on that service. We required that members with a prescription also received a professional service to mitigate selection of individuals based on whether they received professional services. We excluded members from the sample if there were different locations or differences in their demographic data reported across their services or prescriptions. We then selected the subset of members from each treated state and the relevant control states for each cohort.

For example, Connecticut's legislation became effective in July 2014, and we identified Massachusetts as a control because it borders Connecticut and had not passed legislation granting NPs independence. We then identified 4,827,214 continuously enrolled members (across all states) ages 18-64 who had primary medical and prescription drug coverage every month between January 2012 to December 2017. Of these, 4,215,428 had a professional service and consistent location and demographic information. We selected the subset of 17,829 members from Connecticut and 228,870 members from Massachusetts.

[i] Claims are reported on the ZIP level, and we determined the state and county using the ZIP-to-county crosswalk made available by BCBSA.

© 2024 Cusimano LD et al. *JAMA Health Forum.*

eFigure 1. Percent of Total State Population Covered by Final BCBS Axis Analysis Sample

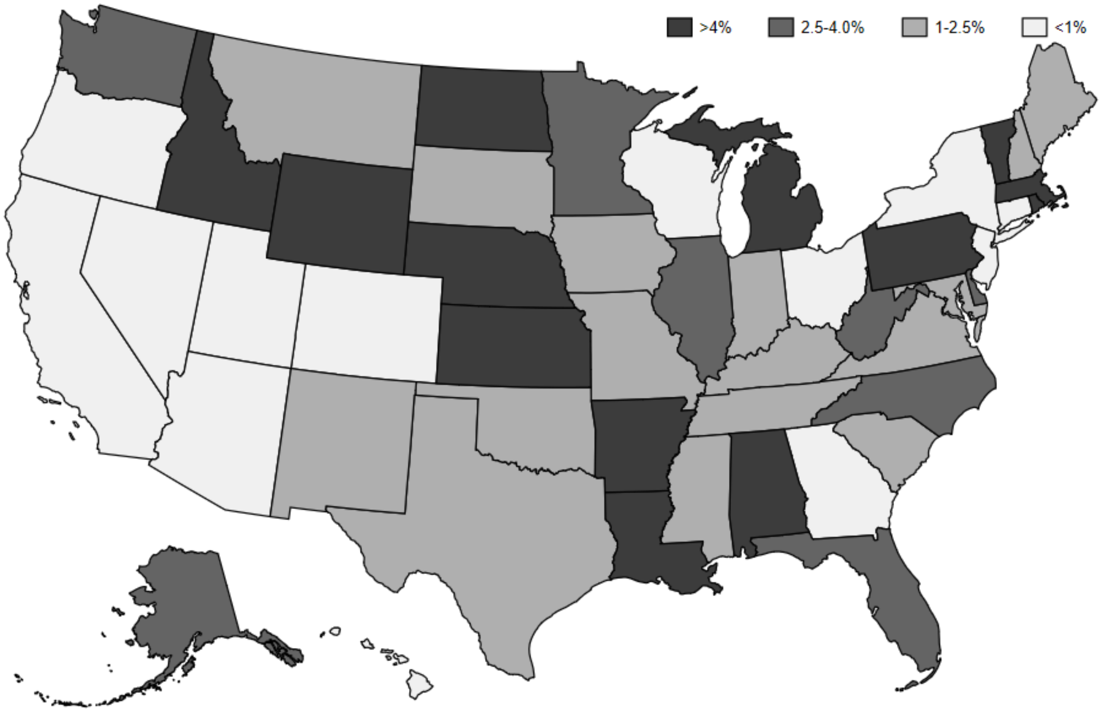

Data range from .08 (Hawaii) to 16.21 (North Dakota)

© 2024 Cusimano LD et al. *JAMA Health Forum.*

eFigure 2. Validation of BCBS Opioid Prescribing Data with CDC Opioid Prescribing Data

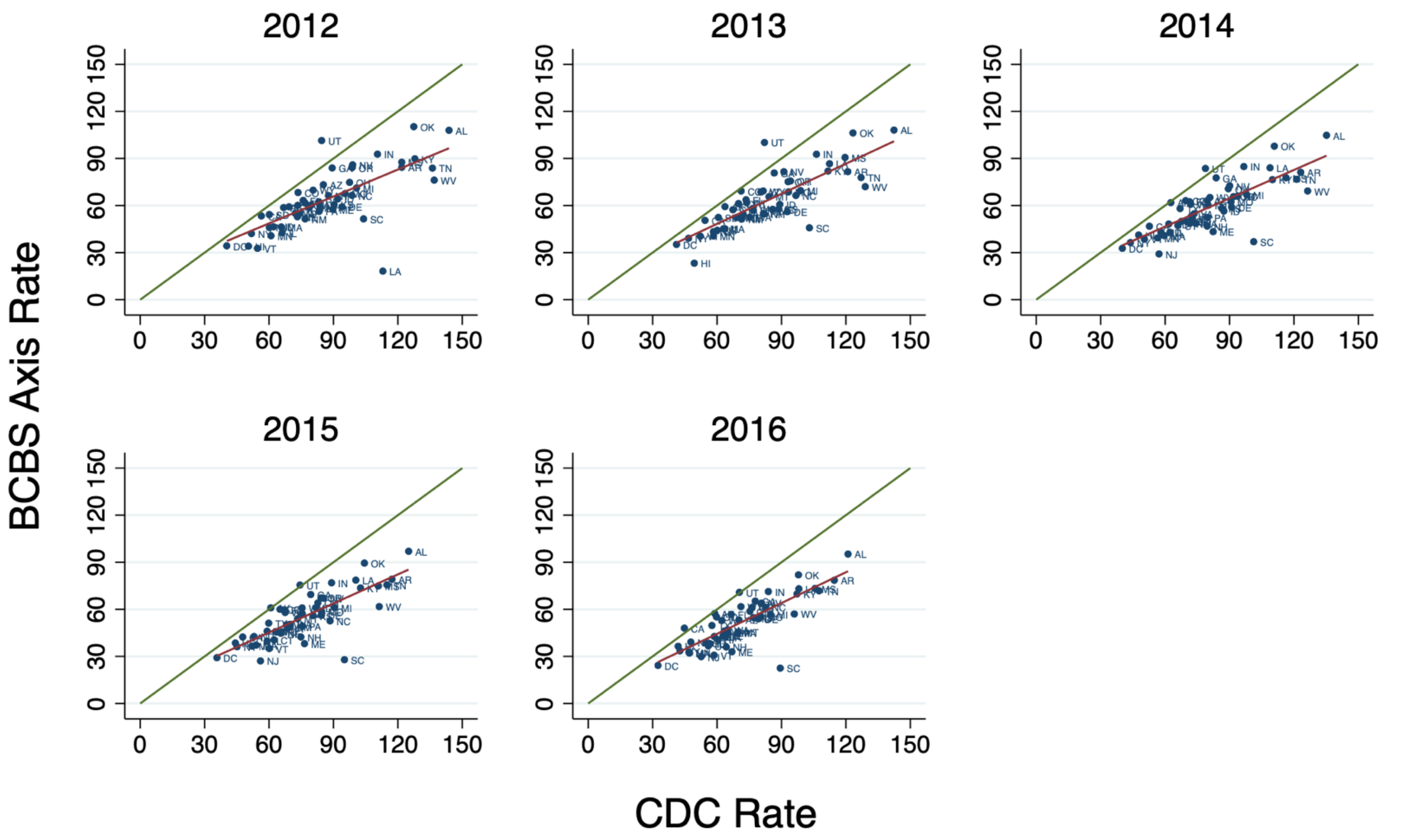

Note. CDC Data taken from the U.S. Opioid Dispensing Rate Tables.[5] "BCBSA" denotes the Blue Cross Blue Shield Association. Red line represents line of best fit. Green line represents y = x.

© 2024 Cusimano LD et al. *JAMA Health Forum.*

eFigure 3. Classification of Counties into Bordering Controls

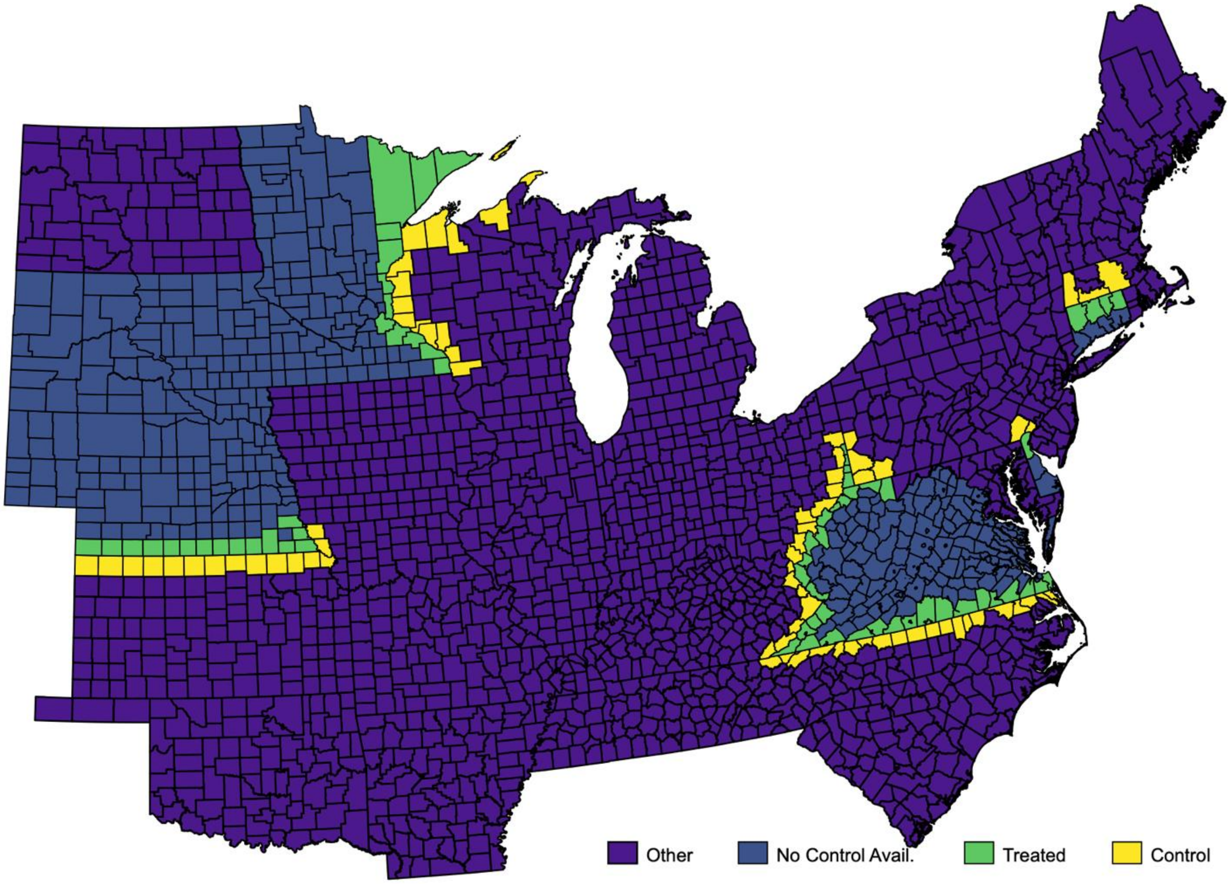

© 2024 Cusimano LD et al. *JAMA Health Forum.*

eFigure 4. Examples of Opioid Prescribing Data for County-level Cohorts

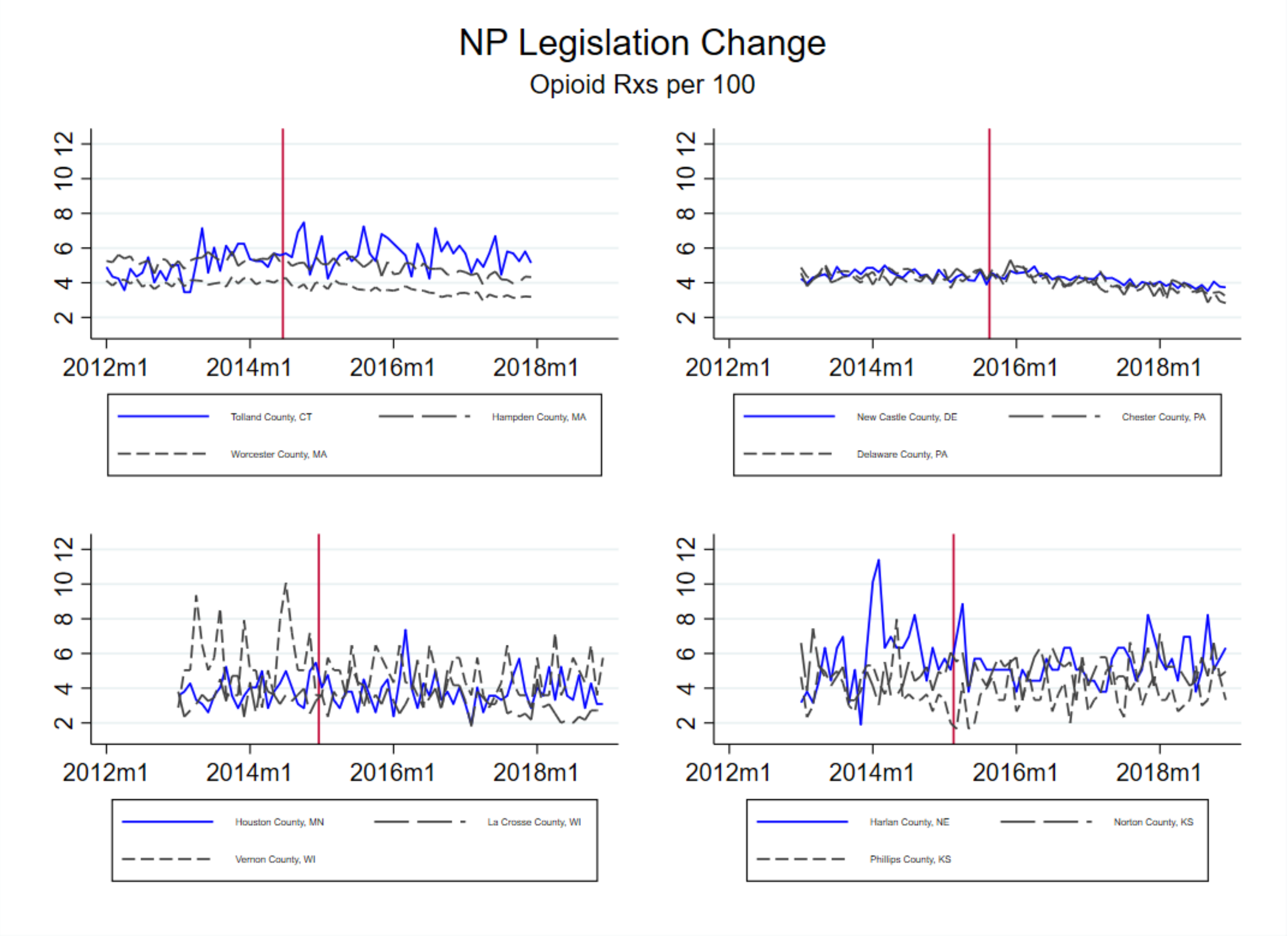

Note. Solid blue line represents the rate for a given county in treated state. Gray lines represent the rate for counties in control states. Solid red line represents the month of legislation going into effect. State abbreviations are: “CT” for Connecticut, “MA” for Massachusetts, “DE” for Delaware, “PA” for Pennsylvania, “MN” Minnesota, “WI” for Wisconsin, "NE" for Nebraska, and “KS” for Kansas.

eFigure 5. Timeline of PDMP Must-Access Laws Relative to NP Legislation

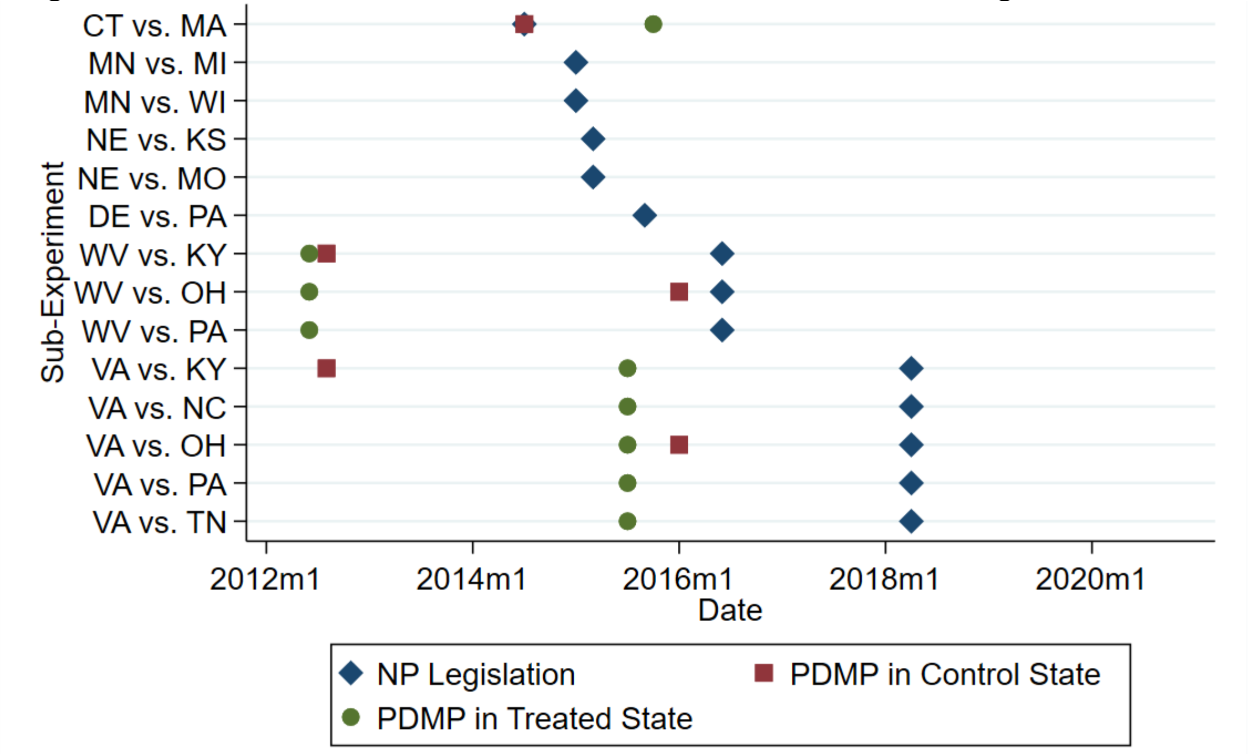

Note. Solid blue line represents the rate for a given county in treated state. Gray lines represent the rate for counties in control states. Solid red line represents the month of legislation going into effect. State abbreviations are: “CT” for Connecticut, “MA” for Massachusetts, “DE” for Delaware, “PA” for Pennsylvania, “MN” Minnesota, “WI” for Wisconsin, "NE" for Nebraska, and “KS” for Kansas. Only PDMP laws present by 2017 are included.

© 2024 Cusimano LD et al. *JAMA Health Forum.*

eTable 1. Construction of Cohorts with Member Counts and Observation Counts

**Treated States**	Connecticut	Delaware	Minnesota	Nebraska	West Virginia	Virginia
**Control States**	Massachusetts	Pennsylvania	Wisconsin, Ohio	Kansas, Missouri	Pennsylvania, Ohio, Kentucky	North Carolina, Kentucky, Tennessee
**Legislation Date**	July 2014	September 2015	January 2015	March 2015	June 2016	April 2018
Six-Year Window	2012-2017	2013-2018			2014-2019	2016-2021
**Counts of Individuals:**						
Coverage in January of first year	53,073,124	55,871,019			60,127,768	69,856,604
Prescription Coverage	26,439,772	28,706,693			31,868,698	37,588,696
Med Coverage	47,073,177	49,576,358			53,289,672	60,954,529
Both	23,794,641	25,839,071			28,572,380	32,886,346
18-64 years old	17,745,149	19,330,140			21,578,369	25,265,616
Unique Members	11,330,957	13,790,210			16,731,077	23,766,837
Continuous 6-Year Enrollment	4,827,214	5,320,671			5,885,931	7,047,853
Received any professional service and consistent location/demographics	4,215,428	4,610,311			5,063,826	5,998,438
In Treated State	17,829	14,750	117,047	75,168	44,326	111,546
In Control State(s)	228,870	385,625	308,168	179,931	484,399	906,554
Total	246,699	400,375	425,215	255,099	528,725	1,018,100
**Aggregation to State-Month Level:**						
Number of Control States	1	1	2	2	3	3
Treated Observations	48	48	48	48	48	48
Control Observations	48	48	96	96	144	144
Problematic Observations	0	0	0	0	0	48
Total Observations	48+48+48+48+48+96+48+96+48+144+48+144-48=816					

© 2024 Cusimano LD et al. *JAMA Health Forum.*

Robustness to Alternative Specifications

eTable 2. Difference-in-Differences Estimated using Alternative Model Specifications

	Opioid Prescriptions					More than 7 days' supply		More than 50 MME per day	
	All	Overlap	Overlap days	Days' supply	MMEs	All	Naive	All	Naive
Base Model	0.00	-0.03	-0.52	-0.16	-0.22	-0.02	0.02	-0.02	0.01
Date FEs (instead of Date-Cohort)	-0.01	-0.03	-0.47	-0.17	-0.27	-0.03	0.01	-0.03	0.00
State FEs (instead of State-Cohort)	0.00	-0.03	-0.52	-0.16	-0.22	-0.02	0.02	-0.02	0.01
Do not restrict to 24 months before/after	0.05	-0.03	-0.66	-0.10	0.04	0.01	0.02	0.01	0.01
Keep outlier data (e.g. Tennessee)	0.10	0.00	0.11	-0.08	-0.20	0.05	0.02	0.01	0.01
Covariates	0.02	-0.02	-0.38	-0.18	-0.30	-0.01	0.02	-0.02	0.01
Outcome Mean	4.9	0.7	10.9	16.0	43.7	2.8	0.3	1.1	0.3
Average % Change over Mean	1%	-3%	-4%	-1%	0%	0%	6%	-1%	2%

Abbreviation: MME, morphine milligram equivalents
Base model uses state-by-cohort and month-by-cohort fixed effects, with errors clustered at the state level.
Covariates: Unemployment rate, average annual pay, number of doctors relative to population, number of NPs relative to population.
* p<0.10, ** p<0.05, *** p<0.01.

© 2024 Cusimano LD et al. *JAMA Health Forum.*

eTable 3. Difference-in-Differences Estimates of Opioid Prescription Outcomes around NP Independence Split by NP Exposure

	Opioid Prescriptions					More than 7 days' supply		More than 50 MME per day	
	All	Overlap	Overlap days	Days' supply	MMEs	All	Naive	All	Naive
Border Controls	0.00	-0.03	-0.52	-0.16	-0.22	-0.02	0.02	-0.02	0.01
SEs	(0.06)	(0.04)	(0.72)	(0.10)	(0.29)	(0.05)	(0.01)	(0.03)	(0.01)
Exposure to NPs	0.02	0.06	1.04	-0.14	-0.62	0.00	0.02	-0.03	-0.01
SEs	(0.08)	(0.09)	(1.59)	(0.19)	(0.45)	(0.07)	(0.02)	(0.06)	(0.02)
Only Physicians	-0.02	-0.05	-0.50	-0.04	-0.31	-0.01	0.02	-0.04	0.00
SEs	(0.07)	(0.04)	(0.71)	(0.12)	(0.38)	(0.06)	(0.01)	(0.04)	(0.02)
Outcome Avg	4.9	0.7	10.9	16.0	43.7	2.8	0.3	1.1	0.3
% Change for Exposure to NPs	0%	8%	10%	-1%	-1%	0%	8%	-3%	-3%

Abbreviation: SE, standard error; MME, morphine milligram equivalents; Avg, average; NP, nurse practitioner.
Standard errors clustered at the state level; state-by-cohort and month-by-cohort fixed effects.
* p<0.10, ** p<0.05, *** p<0.01.

© 2024 Cusimano LD et al. *JAMA Health Forum.*

eTable 4. Difference-in-Differences Estimated using Alternative Control Groups

	Opioid Prescriptions					More than 7 days' supply		More than 50 MME per day	
	All	Overlap	Overlap days	Days' supply	MMEs	All	Naive	All	Naive
Border Controls	0.00	-0.03	-0.52	-0.16	-0.22	-0.02	0.02	-0.02	0.01
County-Border Controls	0.01	-0.01	-0.53	-0.76	0.63	-0.02	0.02	0.00	0.00
All Controls	0.03	-0.04	-0.72	-0.14	0.30	-0.01	0.01	0.02	0.01
Proximate Population (300 m or closest)	-0.03	-0.02	-0.22	-0.16	-0.05	-0.03	0.01	-0.01	0.01
Proximate Population (300 m)	-0.03	0.01	0.21	-0.13	-0.13	-0.03	0.02	-0.02	0.01
Proximate Population (500 m)	-0.01	-0.03	-0.45	-0.12	0.03	-0.03	0.01	-0.02	0.00
Proximate Geography (300 m)	-0.05	-0.01	-0.05	-0.17	0.00	-0.05	0.01	-0.02	0.00
Proximate Geography (500 m)	-0.04	-0.04	-0.61	-0.13	0.04	-0.05	0.01	-0.01	0.01
Outcome Mean	4.9	0.7	10.9	16.0	43.7	2.8	0.3	1.1	0.297
% Change over Mean	0%	-4%	-5%	-1%	-1%	-1%	6%	-2%	2%

Abbreviation: MME, morphine milligram equivalents; m, miles.
State-by-cohort and month-by-cohort fixed effects.; “Proximate Population” refers to distance between the population-weighted centers of the states. “Proximate Geography” refers to the distance between the geographic centers of the states.
* p<0.10, ** p<0.05, *** p<0.01.

© 2024 Cusimano LD et al. *JAMA Health Forum.*

eTable 5. Difference-in-Differences Estimates of Opioid Prescription Outcomes around NP Independence with County Cohorts

	Opioid Prescriptions					More than 7 days' supply		More than 50 MME per day	
	All	Overlap	Overlap days	Days' supply	MMEs	All	Naive	All	Naive
Legislation	0.01	-0.01	-0.53	-0.76	0.63	-0.02	0.02	0.00	0.00
SE	(0.10)	(0.10)	(2.13)	(0.46)	(0.80)	(0.09)	(0.03)	(0.03)	(0.01)
95% CI	[-0.21,0.22]	[-0.22,0.19]	[-5.10,4.03]	[-1.74,-0.23]	[-0.47,1.72]	[-0.21,0.17]	[-0.03,0.08]	[-0.07,0.07]	[-0.03,0.03]
Outcome avg	5.0	0.8	13.8	16.3	41.6	3.0	0.3	1.0	0.3
Change from avg	0%	-2%	-4%	-5%	2%	-1%	9%	0%	1%
N obs	9504	9504	9504	9378	9378	9504	9504	9504	9504
Adj R-sq	0.6	0.6	0.6	0.5	0.7	0.7	0.1	0.4	0.0
F	0.0	0.0	0.1	2.7	1.5	0.1	1.0	0.0	0.0

Abbreviation: MME, morphine milligram equivalents; SE, standard error; CI, confidence interval; Avg, average; N obs, number of observations; Adj R-sq, adjusted R-squared.
Standard errors in parentheses, clustered at the state level; state-by-cohort and month-by-cohort fixed effects.
* p<0.10, ** p<0.05, *** p<0.01.

© 2024 Cusimano LD et al. *JAMA Health Forum.*

eTable 6. Difference-in-Differences Estimates of Opioid Prescription Outcomes around NP Independence Controlling for Must-Access PDMP Legislation

	Opioid Prescriptions					More than 7 days' supply		More than 50 MME per day	
	All	Overlap	Overlap days	Days' supply	MMEs	All	Naive	All	Naive
NP Legislation	-0.02	-0.04	-0.76	-0.18	-0.33	-0.04	0.02	-0.03	0.00
SE	(0.07)	(0.04)	(0.82)	(0.12)	(0.31)	(0.06)	(0.01)	(0.03)	(0.01)
PDMP active in:									
Control state	-0.14	-0.06	-1.10	0.03	-0.42	-0.11	-0.01	-0.06	-0.02
	(0.14)	(0.04)	(0.79)	(0.16)	(0.17)	(0.10)	(0.03)	(0.02)	(0.02)
Treated state	-0.14	0.00	0.80	0.56	0.73	-0.02	0.00	0.02	0.00
	(0.07)	(0.02)	(0.22)	(0.07)	(0.10)	(0.05)	(0.02)	(0.01)	(0.01)
Outcome avg	4.9	0.7	10.9	16.0	43.7	2.8	0.3	1.1	0.3
Change from avg	0%	-6%	-7%	-1%	-1%	-1%	5%	-3%	0%

Abbreviation: MME, morphine milligram equivalents; SE, standard error; CI, confidence interval; Avg, average; N obs, number of observations; Adj R-sq, adjusted R-squared.
Standard errors in parentheses, clustered at the state level; state-by-cohort and month-by-cohort fixed effects. Controls for whether a must-access Prescription Drug Monitoring Program (PDMP) was in place in the treated and control states were included.
* p<0.10, ** p<0.05, *** p<0.01.

© 2024 Cusimano LD et al. *JAMA Health Forum.*

eReferences

1. FDA. National Drug Code Directory. FDA. May 10, 2022. Accessed June 6, 2022. https://www.fda.gov/drugs/drug-approvals-and-databases/national-drug-code-directory

2. CDC. Data Resources: CDC's Response to the Opioid Overdose Epidemic. October 18, 2021. Accessed June 6, 2022. https://www.cdc.gov/opioids/data-resources/index.html

3. FDA. *FDA Drug Safety Communication: FDA Warns about Serious Risks and Death When Combining Opioid Pain or Cough Medicines with Benzodiazepines; Requires Its Strongest Warning*. FDA; 2016. Accessed May 26, 2022. https://www.fda.gov/drugs/drug-safety-and-availability/fda-drug-safety-communication-fda-warns-about-serious-risks-and-death-when-combining-opioid-pain-or

4. Goodman-Bacon A. Difference-in-differences with variation in treatment timing. *Journal of Econometrics*. 2021;225(2):254-277. doi:10.1016/j.jeconom.2021.03.014

5. CDC. U.S. Opioid Dispensing Rate Maps. November 10, 2021. Accessed April 13, 2023. https://www.cdc.gov/drugoverdose/rxrate-maps/index.html

© 2024 Cusimano LD et al. *JAMA Health Forum.*
