# Supplementary material for: High-Risk Opioid Prescribing and Nurse Practitioner Independence
Source: JAMA Health Forum. 2024 Dec 20;5(12):e244544. doi: 10.1001/jamahealthforum.2024.4544 (PMC11662257; doi:10.1001/jamahealthforum.2024.4544)
Supplement: Supplement 2. — Data sharing statement [file jamahealthforum-e244544-s002.pdf]

Data Sharing Statement

Cusimano. High-Risk Opioid Prescribing and Nurse Practitioner Independence. *JAMA Health Forum*. Published December 20, 2024. doi:10.1001/jamahealthforum.2024.4544

Data
Data available: Yes
Data types: Deidentified participant data, Data (not involving human participants)
How to access data: Aggregated data and analytic code are available upon request from the corresponding author (Lucas.D.Cusimano.MED@Dartmouth.edu) and with permission of the data partner, Blue Cross Blue Shield (BCBS) Axis.
When available: With publication

Supporting Documents
Document types: Statistical/analytic code
How to access documents: Aggregated data and analytic code are available upon request from the corresponding author (Lucas.D.Cusimano.MED@Dartmouth.edu) and with permission of the data partner, Blue Cross Blue Shield (BCBS) Axis.
When available: With publication

Additional Information
Who can access the data: Researchers whose proposed use of the data has been approved by BCBS Axis.
Types of analyses: Replication, additional analyses, and/or other work that could reasonably be carried out using the data.
Mechanisms of data availability: Investigators will pass along requests for data and code to BCBS Axis.
Any additional restrictions: All uses of the data must be approved by BCBS Axis. There should be no attempt to identify individuals in the data.
